# Multifocal/multicentric breast cancer: Does each focus matter?

**DOI:** 10.1002/cam4.5626

**Published:** 2023-02-03

**Authors:** Ying Tong, Feixiang Sun, Chuanpeng Zhang, Susu Yang, Ziyi Yu, Yi Zhao

**Affiliations:** ^1^ Jiangsu Breast Disease Center Jiangsu Province Hospital and Nanjing Medical University First Affiliated Hospital Nanjing China

**Keywords:** breast cancer, intertumoral heterogeneity, multicentrical, multifocal, tumor stage

## Abstract

**Background:**

Multifocal (MF) and multicentric (MC) breast cancer cases have been increasingly diagnosed owing to the extensive use of improved preoperative breast imaging. The current tumor‐node‐metastasis staging system uses the dimension of the largest tumor and recommends reporting the pathological features of the largest tumor in MF/MC breast cancers.

**Aim:**

This study aimed to explore whether the largest or aggregate dimensions of MF and MC breast cancers can better predict tumor behavior. We also attempted to study the histological and biological heterogeneities of separate foci in MF and MC breast cancers to determine whether it was necessary to examine each lesion.

**Methods:**

We retrospectively analyzed 121 patients with MF/MC (103 with MF and 18 with MC) breast cancers and 484 patients with unifocal breast cancer who were treated at the First Affiliated Hospital of Nanjing Medical University. Two methods were used to record the T stage (using the dimensions of the largest lesion and aggregate dimensions of all lesions). The histological grade, immunohistochemical parameters, and molecular subtypes of the largest lesion and other lesions in MF/MC breast cancers were studied to assess intertumoral heterogeneity.

**Results:**

The use of aggregate dimensions upstaged 63 patients with MF/MC breast cancers to a more advanced stage and removed the independent effect of cancer multiplicity on lymph node positivity compared with the use of the largest dimension. Mismatches were found in the pathological type (9.9%), histological grade (4.1%), and molecular subtype (8.3%) among different foci.

**Conclusion:**

The tendency of MF/MC breast tumors to metastasize may be related to tumor load, which can be better predicted by the aggregate dimensions of all foci. The use of the current staging systems may require further evaluation and modification. Intertumoral heterogeneity indicates the necessity for pathological and immunohistochemical assessments of each lesion in patients with MF/MC breast cancers.

## Graphical abstract

Lawsone inhibits the proliferation and metastasis of residual breast cancer cells after microwave ablation by inducing cellular inhibitory autophagy through NCAPG/AURKB/AKT/mTOR axis.

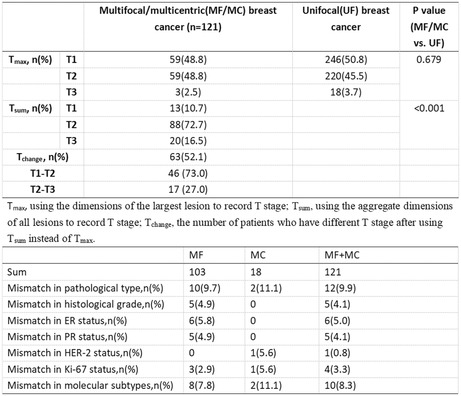

## 1 INTRODUCTION

Breast cancer is the most common malignant cancer worldwide, and its incidence rate increases annually.^1^ Preoperative breast imaging, especially magnetic resonance imaging (MRI), has been extensively used in recent years. Hence, multifocal (MF) and multicentric (MC) breast cancer cases are being increasingly diagnosed.^2^, ^3^ Generally, MF and MC breast cancers are defined as two or more separate foci in the same quadrant or in different quadrants of the same breast respectively.^4^, ^5^ However, the definition of quadrant was not presented in the majority of previous studies, and the definition of MF and MC breast cancers remains controversial.^6^ Owing to the lack of a standard definition and the different diagnostic methods, the incidence rates of multiple tumors with a wide range of 9–75% have been reported.^7^

Tumor size is a significant predictor of lymph node metastasis and can affect the final survival outcomes.^8^, ^9^ The current tumor‐node‐metastasis (TNM) staging system and the 8th edition of the American Joint Committee on Cancer staging manual only use the dimension of the largest tumor and recommend the report of histological grade, pathological type, and other pathological features of the largest tumor in MF/MC breast cancers.^10^, ^11^ These guidelines consider that prognosis mainly depends on the largest tumor, overlooking the total tumor load and heterogeneity of different lesions.

While formulating treatment plans such as radiotherapy and chemotherapy, tumor size should not be neglected.^12^ It is important to determine whether using the dimension of the largest tumor for T staging would underestimate the real tumor size and load to influence the selection of correct treatment.^13^ Some scholars have demonstrated that the use of aggregate dimensions of all foci may accurately predict tumor behavior in MF/MC breast cancers.^5^, ^14^ Moreover, several studies have highlighted the importance of independent assessment and reporting of each lesion because the treatment strategy and prognostic outcome can also be influenced by intertumoral heterogeneity.^13^, ^15^, ^16^, ^17^

Only few studies have focused on MF/MC breast cancers, which leads to the neglect of the particularity of these patients. According to some researches, determining how to stage tumors and whether to assess all lesions were contested.^15^, ^16^, ^18^, ^19^ These can guide clinical evaluation and enable clinicians to formulate more accurate and comprehensive therapy plans for patients with MF/MC breast cancers. Hence, in this study, we attempted to explore whether the largest or aggregate dimensions could better predict tumor behavior. Moreover, we would like to observe the histological and biological heterogeneities of different lesions in MF and MC breast cancers to study the necessity of examining each lesion to guide clinicians in the treatment of MF and MC breast cancers.

## 2 MATERIALS AND METHODS

### 2.1 Patients' selection

In the present study, the medical records of patients with unifocal (UF), MF, and MC breast cancers who were admitted to the First Affiliated Hospital of Nanjing Medical University from January 2010 to December 2016 and from September 2020 to June 2022 were retrospectively analyzed. MF was defined as two or more separate foci in the same quadrant, whereas MC was defined as two or more separate foci in different quadrants of the same breast. All patients underwent MRI examination before surgery to screen patients suspected of having MF/MC breast cancers. The final diagnosis was established based on pathology. The main lesion should be invasive, whereas the other lesion could be invasive or carcinoma in situ when it was confirmed as an independent lesion by both pathology and imaging. We defined two foci as being in the same quadrant when they were connected to the nipple at an angle of <90°. These foci were separated from each other by uninvolved breast tissue including normal tissue or benign lesions, regardless of the distance between foci.^20^ Patients who previously had breast cancer or other types of cancer, multiple carcinomas in situ, and distant metastases and those who received neoadjuvant treatment and male were excluded from this study. Patients who were suspected to have MF/MC breast cancers by preoperative imaging and confirmed by pathology after surgery and did not meet any of the above exclusion criteria were included in this study. Patients with UF breast cancer were randomly selected via matching with patients with MF and MC breast cancers according to immunochemical type and menopausal status in a 1:4 ratio (MF/MC: UF). In total, 605 patients (103, 18, and 484 patients with MF, MC, and UF breast cancers, respectively) were included in the present study.

### 2.2 Data collection and evaluation

Clinicopathological data, such as age, menopausal status, lesion size, number of lesions, lymph node status, histological grade, lymphovascular/perineural invasion, pathological type, surgery, immunohistochemical parameters (including estrogen receptor [ER], progesterone receptor [PR], human epidermal growth factor receptor 2 [HER‐2], Ki‐67), fluorescence in situ hybridization (FISH), and molecular subtypes (luminal subtype), were obtained from electronic medical records or pathological data.

A threshold of 1% stained breast cancer cells was used to define ER‐ or PR positivity.^21^ HER‐2 was scored as 0, 1+, 2+, or 3+, and a staining score of 3+ was defined as positive. Tumors with a 2+ score were retested using FISH to determine whether the HER‐2 gene was amplified.^22^ Ki‐67 staining was labeled as “low proliferation” with a positive staining of ≤14% and as “high proliferation” with a positive staining of >14%. Molecular subtypes were classified as luminal A, luminal B, HER‐2‐positive, and triple‐negative based on the immunochemistry results of ER, PR, HER‐2, and Ki‐67 as follows: luminal A subtype (ER‐positive and PR > 20% positive, HER‐2‐negative, and low proliferation), luminal B subtype (HER‐2‐positive) (ER‐positive and/or PR > 20% positive, and HER‐2‐positive, and/or high proliferation), and luminal B subtype (HER‐2‐negative) (ER‐positive, HER‐2‐negative, at least one of the following criteria: PR < 20% positive or high proliferation), HER‐2‐positive (ER‐negative, PR‐negative, and HER‐2‐positive), and triple‐negative subtype (ER‐negative, PR‐negative, and HER‐2‐negative).^23^

According to the 8th edition of the TNM staging guidelines, only the largest tumor is considered when recording the T stage and multiplicity is indicated using the suffix(m).^10^ In the present study, two methods were used to record the T stage: using the dimension of the largest invasive tumor (T_max_ stage) and the aggregate dimensions of all invasive foci (T_sum_ stage). The pathological characteristics of the largest invasive tumor were recorded, and the characteristics of the other lesions were reviewed to assess intertumoral heterogeneity. Mismatches in PR, ER, and HER‐2 were defined when at least one of the lesions' positive or negative results was different from that of other lesions. At least one lesion of “high proliferation” with other lesions of “low proliferation” was defined as a mismatch in Ki‐67.

### 2.3 Statistical analyses

Statistical analyses were performed using the Statistical Package for the Social Sciences version 25 software (IBM). MC and MF tumors were compared with UF tumors as a group (MF/MC). Categorical variables were compared using contingency tables and the chi‐squared or two‐tailed Fisher's exact test. Continuous variables were investigated using the Kolmogorov–Smirnov test to determine whether they were normally distributed. Abnormally distributed continuous variables were analyzed using a nonparametric test (Mann–Whitney *U* test). Factors significantly associated with lymph node positivity were evaluated using univariate and multivariate logistic regression analyses. Differences were considered statistically significant at *p* ≤ 0.05.

## 3 RESULTS

### 3.1 Multifocal and Multicentric breast cancers were more aggressive than unifocal breast cancer

In total, 103 patients with MF breast cancer (87 patients had 2 foci, 11 had 3 foci, 3 had 4 foci, 1 had 5 foci, and 1 had 7 foci) and 18 patients with MC breast cancer (9 patients had 2 foci, 8 had 3 foci, and 1 had 4 foci) were enrolled in this study. Among these 121 patients, 22 (18.2%) had luminal A subtype, 70 (57.9%) had luminal B subtype, 21 (17.4%) had HER‐2 positivity, and 8 (6.6%) had triple‐negative subtype. There were 71 (58.7%) premenopausal and 50 (41.3%) postmenopausal breast cancer cases. A total of 484 patients with UF breast cancer were identified in a 1:4 ratio with patients with MF/MC breast cancer.

The clinicopathological characteristics of the MF, MC, and UF groups are shown in Table 1. No significant difference was observed after comparing UF with MF/MC in terms of age, T_max_ stage, and pathological type. Patients in the MF and MC groups were more likely to have histological grade 3 (MF/MC and UF groups, 64.5% and 53.7%, respectively; *p* = 0.033), lymph node metastases (MF/MC and UF groups, 53.7% and 37.2%, respectively; *p* = 0.001) and lymphovascular/perineural invasion (MF/MC and UF groups, 32.2% and 18.2%, respectively; *p* = 0.001) than patients in the UF group. Patients with UF breast cancer preferred to undergo breast‐conserving surgery over mastectomy (MF/MC and UF groups, 13.2% and 47.3%, respectively; *p* < 0.001).

**TABLE 1:** Clinical and pathological characteristics of patients with multifocal (MF), multicentric (MC), and unifocal (UF) breast tumors.

	MF	MC	MF/MC	UF	*p* value (MF/MC versus UF)
Number, *n*	103	18	121	484	
Age, *n* (%)					
≤50	66 (64.1)	8 (44.4)	74 (61.2)	274 (56.6)	0.366
>50	37 (35.9)	10 (55.6)	47 (38.8)	210 (43.4)	
T_max_, *n* (%)					
T1	52 (50.5)	7 (38.9)	59 (48.8)	246 (50.8)	0.679
T2	48 (46.6)	11 (61.1)	59 (48.8)	220 (45.5)	
T3	3 (2.9)	0 (0)	3 (2.5)	18 (3.7)	
Tumor diameter (the largest tumor) (mm)	23.86 (1.4–80)	26.28 (10–43)	24.22 (1.4–80)	23.40 (2.5–90)	0.837
T_sum_, *n* (%)					
T1	13 (12.6)	0 (0)	13 (10.7)		<0.001
T2	76 (73.8)	12 (66.7)	88 (72.7)		
T3	14 (13.6)	6 (33.3)	20 (16.5)		
Tumor diameter (sum) (mm)	36.70 (2.5–96)	46.06 (25–75)	38.09 (2.5–96)		<0.001
Lymph node positivity, *n* (%)					
Positive	53 (51.5)	12 (66.7)	65 (53.7)	180 (37.2)	0.001
Negative	50 (48.5)	6 (33.3)	56 (46.3)	304 (62.8)	
Histological grade (misssing, 22), *n* (%)					
I–II	35 (34.0)	4 (22.2)	39 (32.2)	206 (42.6)	0.033
III	68 (66.0)	14 (77.8)	78 (64.5)	260 (53.7)	
Lymphovascular/perineural invasion, *n* (%)					
(+)	31 (30.1)	8 (44.4)	39 (32.2)	88 (18.2)	0.001
(−)	72 (69.9)	10 (55.6)	82 (67.8)	396 (81.8)	
Pathological type, *n* (%)					
IDC	95 (92.2)	15 (83.3)	110 (90.9)	445 (91.9)	0.879
ILC	1 (1.0)	1 (5.6)	2 (1.6)	9 (1.9)	
Other	7 (6.8)	2 (11.1)	9 (7.4)	30 (6.2)	
Surgery, *n* (%)					
Mastectomy	87 (84.5)	18 (100)	105 (86.8)	255 (52.7)	<0.001
BCS	16 (15.5)	0 (0)	16 (13.2)	229 (47.3)	

Abbreviations: BCS, Breast conserving surgery; DCIS, Ductal carcinoma in situ; IDC, Invasive ductal carcinoma; ILC, Invasive lobular carcinoma.

### 3.2 Using the aggregate dimensions of all foci elevated T stage of 63 patients and removing the independent effect of cancer multiplicity on lymph node positivity

First, we followed the current TNM staging system and identified 59 (48.8%) patients in the T1 stage, 59 (48.8%) in the T2 stage, and 3 (2.5%) in the T3 stage. The staging method was adjusted to use the aggregate dimensions of all the lesions. We found that staging changed in 63 (52.1%) patients, of whom 46 (73.0%) changed from T1 to T2, and 17 (27.0%) changed from T2 to T3. Overall, 13 (10.7%) patients were in the T1 stage, 88 (72.7%) were in the T2 stage, and 20 (16.5%) were in the T3 stage. There were more patients with MF/MC breast cancer in the T2 and T3 stages after adjusting for the staging method.

When T_max_ was used for staging of patients with MF/MC breast cancer, the rate of lymph node metastasis was similar in the MF/MC and UF groups in the T_max_2 and T_max_3 stages. Patients with MF/MC breast cancer in T_max_1 stage had more lymph node positivity (44.1% vs. 25.2%, *p* = 0.004) than patients with UF breast cancer. However, when using T_sum_ stage, the difference in lymph node positivity rates between the MF/MC and UF groups was no longer observed (Table 2).

**TABLE 2:** Lymph node positivity in different T stages in patients with MF and MC breast tumors using different measurement methods and with UF breast tumors.

	Lymph node positivity (%)		*p* value
	MF/MC	UF	
T stage (using the largest tumor diameter)			
T1	26/59 (44.1)	62/246 (25.2)	0.004
T2	37/59 (62.7)	109/220 (49.6)	0.072
T3	2/3 (66.7)	9/18 (50.0)	1.000
T stage (using the aggregate tumor diameter)			
T1	3/13 (23.1)	62/246 (25.2)	1.000
T2	46/88 (52.3)	109/220 (49.6)	0.665
T3	16/20 (80.0)	9/18 (50.0)	0.087

Factors related to lymph node positivity were also investigated. Patients with MF/MC breast cancer, lymphovascular/perineural invasion, high histological grade, and T stage (both T_max_ and T_sum_) were more likely to have lymph node metastases in the univariate analysis (Table 3). Statistically significant factors in the univariate analysis were assessed using a multivariate logistic regression model. Lymphovascular/perineural invasion positivity (*p* < 0.001) and high T stage (both T_max_ and T_sum_) (*p* < 0.001) were independent factors of lymph node metastasis. Importantly, we found that cancer multiplicity was an independent factor affecting lymph node status when using the largest dimension to define T stage, whereas when using the aggregate dimensions, cancer multiplicity was no longer associated with lymph node positivity (*p* = 0.016 for T_max_, *p* = 0.559 for T_sum_) (Table 4).

**TABLE 3:** Univariate analysis of lymph node positivity.

	Lymoh node positivity, *n* (%)	Univariable analysis		
		Odds ratio	95% Cl for odds ratio	*p* value
Pathological type				0.941
IDC	224/555 (40.4)	Referent		
ILC	5/11 (45.5)	1.231	0.371–4.084	0.734
Other	16/39 (41.0)	1.028	0.531–1.989	0.935
Lymphovascular/perineural invasion				
(−)	158/478 (33.1)	Referent		
(+)	105/127 (82.7)	4.405	2.894–6.705	<0.001
Histological grade				
I–II	81/245 (33.1)	Referent		
III	160/338 (47.3)	1.809	1.285–2.545	0.001
T_max_ stage (using the largest tumor diameter)				<0.001
T1	88/305 (28.9)	Referent		
T2	146/279 (52.3)	2.512	1.788–3.529	<0.001
T3	11/21 (52.4)	3.185	1.297–7.823	0.012
T_sum_ stage(using the aggregate tumor diameter)				<0.001
T1	65/259 (25.1)	Referent		
T2	155/308 (50.3)	3.103	2.167–4.443	<0.001
T3	25/38 (65.8)	4.576	2.253–9.295	<0.001
Multiplicity				
UF	180/484 (37.2)	Referent		
MF/MC	65/121 (53.7)	1.960	1.311–2.931	0.001

**TABLE 4:** Multivariate analysis of lymph node positivity.

	Multivariable analysis					
	Using the largest tumor diameter			Using the aggregate tumor diameter		
	Odds ratio	95% Cl for odds ratio	*p* value	Odds ratio	95% Cl for odds ratio	*p* value
Lymphovascular/perineural invasion						
(+) versus (−)	3.723	2.390–5.800	<0.001	3.667	2.352–5.716	<0.001
Histological grade						
III versus I–II	1.202	0.826–1.749	0.337	1.210	0.831–1.761	0.321
T stage			<0.001			<0.001
T2 versus T1	2.290	1.581–3.316	<0.001	2.643	1.776–3.934	<0.001
T3 versus T1	2.458	0.946–6.386	0.065	3.193	1.454–7.009	0.004
Multiplicity						
Multifocal versus unifocal	1.722	1.107–2.681	0.016	1.149	0.722–1.929	0.559

### 3.3 Mismatches among different foci were found in pathological type, histological grade, estrogen receptor, progesterone receptor, human epidermal growth factor receptor 2, Ki‐67, and molecular subtype

We reviewed the pathology reports of both the largest lesion and other lesions in patients with MF/MC breast cancer. Mismatches among different foci were also found in pathological type (9.9%), histological grade (4.1%), ER (5.0%), PR (4.1%), HER‐2 (0.8%), Ki‐67 (3.3%), and molecular subtype (8.3%) (Table 5). Twelve patients had different pathological lesion types. The main lesion in 10 patients were invasive ductal cancer (IDC), followed by ductal carcinoma in situ (DCIS). One patient had an invasive papillary carcinoma with intraductal papillary carcinoma. One patient had intracystic papillary carcinoma with IDC. The histological grade differed among five patients. The histological grade of the largest lesion in one patient was lower than that of the other lesions. In addition, six patients had a mismatch in ER, of whom three patients had ER positivity in the other lesions, while ER negativity was found in the largest lesion. One patient had a difference in HER‐2 status, in which the largest lesion was found to be HER‐2‐negative and the other was HER‐2‐positive.

**TABLE 5:** Heterogeneity of different parameters in MF and MC breast tumors.

	MF	MC	MF + MC
Sum	103	18	121
Mismatch in pathological type, *n* (%)	10 (9.7)	2 (11.1)	12 (9.9)
Mismatch in histological grade, *n* (%)	5 (4.9)	0	5 (4.1)
Mismatch in ER status, *n* (%)	6 (5.8)	0	6 (5.0)
Mismatch in PR status, *n* (%)	5 (4.9)	0	5 (4.1)
Mismatch in HER‐2 status, *n* (%)	0	1 (5.6)	1 (0.8)
Mismatch in Ki‐67 status, *n* (%)	3 (2.9)	1 (5.6)	4 (3.3)
Mismatch in molecular subtypes, *n* (%)	8 (7.8)	2 (11.1)	10 (8.3)

A difference in molecular subtype was found in 10 patients, of whom eight had MF breast cancer and two had MC breast cancer (Table 6). Among these patients, nine had the same histological grade and seven had the same pathological type. One patient with a difference in both histological grade and pathological type had the largest lesion of the luminal B subtype and another lesion of the luminal A subtype. One of the two patients who differed only in pathological type had luminal B subtype (HER‐2‐positive) and HER‐2 positivity, and the other had luminal A and B subtypes.

**TABLE 6:** Clinical characteristics of 10 cases of MF breast cancer with different molecular subtypes.

Case	Focus	Diameter (mm)	Histology	Histological grade	ER	PR	HER‐2	KI‐67 (%)	Molecular subtype	Multiplicity
Case1	7	8,6,6,6,6,4,3	IDC^a^	III^a^	(−)	(−)	(+)	50	Her‐2	MF
					(+)	(−)	(+)	40	Luminal B (HER‐2+)	
Case2	2	20,12	IDC	III^a^	(+)	(+)	(+)	50	Luminal B (HER‐2+)	MF
			IDC + DCIS^b^		(−)	(−)	(+)	50	HER‐2	
Case3	3	30,15	IDC + DCIS	III	(+)	(+)	(−)	40	LuminalB	MF
			Intraductal papillary carcinoma+DCIS^b^	II^b^	(+)	(+)	(−)	12	LuminalA	
Case4	2	20,18	IDC + DCIS^a^	III^a^	(+)	(−)	(+)	50	HER‐2	MF
					(−)	(−)	(+)	60	Luminal B (HER‐2+)	
Case5	2	25,8	IDC^a^	III^a^	(−)	(−)	(−)	80	Triple negative	MF
					(+)	(−)	(−)	60	LuminalB	
Case6	2	20,5	IDC + DCIS^a^	III^a^	(+)	(+)	(−)	15	LuminalB	MF
					(+)	(+)	(−)	8	LuminalA	
Case7	2	26,2	IDC + DCIS^a^	II^a^	(+)	(+)	(−)	40	LuminalB	MF
					(−)	(−)	(−)	50	Triple negative	
Case8	2	30,15	IDC + DCIS^a^	III^a^	(+)	(−)	(+)	30	LuminalB (HER‐2+)	MF
					(−)	(−)	(+)	30	HER‐2	
Case9	3	30,15,10	IDC^a^	III^a^	(−)	(−)	(−)	60	Triple negative	MC
					(−)	(−)	(+)	70	HER‐2	
Case10	2	13,12	Intracystic papillary carcinoma	III^a^	(+)	(+)	(−)	10	LuminalA	MC
			IDC^b^		(+)	(+)	(−)	20	LuminalB	

^a^ Same.

^b^ Different.

## 4 DISCUSSION

With the development of imaging and pathology, the detection rates of MF and MC breast cancers have increased^24^; however, there are still some unsolved challenges. It remains controversial whether the aggressiveness of MF/MC breast cancers is caused by special biological characteristics or high tumor burden.^6^ The current staging systems that only record the dimension and stage of the largest lesion underestimate the tumor burden^25^, ^26^ and neglect intertumoral heterogeneity. In our study, the mean dimensions of the largest lesion and the stage of MF/MC breast cancer were identical to those of UF breast cancer. However, the mean dimensions of all lesions were larger in MF/MC breast cancer than in UF breast cancer. While using the aggregate dimensions of all foci, staging in 63 patients upgraded and more patients with MF/MC breast cancer were in the T2 and T3 stages. Although we only found differences in lymph node positivity in the T1 stage (lesion size <20 mm), there was no difference after adjusting the staging method to use the sum size.

In the univariate and multivariate logistic regression analyses of factors associated with lymph node positivity, which was associated with aggressiveness and poor outcome, lymphovascular/perineural invasion positivity, and high T stage (both T_max_ and T_sum_) were found to be independent factors for lymph node metastases. All of these factors were identified as high‐risk factors that should be considered when making clinical decisions.^27^ Importantly, cancer multiplicity was not found to be an independent risk factor for lymph node metastases after using the sum size to stage. The size of the lesion directly affects the radiotherapy.^12^ In our hospital, patients with tumors ≥5 cm and lymph node metastases are generally recommended to undergo radiotherapy. Using the dimensions of the largest invasive lesion may underestimate the T stage and the possibility of metastasis, which may cause patients to miss the opportunity to undergo radiotherapy. Therefore, we inferred that the aggressiveness of MF/MC breast cancers could be due to the total tumor load, which could be properly predicted by the aggregate dimensions of all invasive foci.^14^, ^25^, ^28^, ^29^

In addition to increasing the tumor load, the other lesions were also found to have heterogeneity with the largest lesion (Tables 5 and 6). Among the five patients with different histological grades, one had a higher grade of another lesion. Molecular subtype‐based differences were found in 10 patients, with the seven patients' treatment possibly changing if we valued each focus. Moreover, in these 10 cases patients, seven had no pathological type or histological grade heterogeneity. Consequently, paying attention to the largest lesion may deprive patients of the opportunity to undergo appropriate therapies (e.g., endocrine and targeted therapies, and chemotherapy). To provide effective treatments, it is essential to fully characterize all lesions, especially the molecular subtype, regardless of the pathological type and histological grade of the largest and additional foci.^15^, ^16^, ^17^, ^30^, ^31^

Fushimi et al.^32^ studied 136 (18.5%) patients with MF/MC breast cancer. After adopting the sum size of each lesion, the T stage of the 36 patients with MF/MC was upstaged. They found that MF/MC was upstaged by the modified T stage, which was associated with worse disease‐free survival than non‐upstaged MF/MC (*p* = 0.004). According to the results of multivariate analysis, upstaged MF/MC was an independent factor for poor prognosis. Coombs et al.^5^ also found that the use of aggregate dimensions reclassified a significant number of MF breast tumors at a more advanced stage and eliminated the association between cancer multiplicity and lymph node positivity. In a subsequent study,^29^ at a median follow‐up time of 10.4 years, for tumors that were >20 mm, using aggregate tumor size eliminated the significant difference in 10‐year survival rate between MF and UF breast tumors (*p* = 0.008 and *p* = 0.49 respectively). Thus, these two studies have demonstrated that the tendency of breast tumors to metastasize is related to the total tumor load and that the use of current guidelines to stage MF breast tumors may require modification.

Onisai et al.^26^ studied 31 patients with MF breast cancer and six with MC breast cancer. Several mismatches between the index and secondary tumors were detected, including 3 (8.1%) patients with histopathological mismatch, 13 (35.1%) with different grades of differentiation, 11 (29.8%) with ER status mismatch, 12 (32.4%) with PR status mismatch, 8 (21.6%) with molecular phenotype mismatch, and 17 (45.9%) with variable Ki‐67 expression levels. Secondary tumors in five patients were dominant, which would cause changes in the therapeutic decision. Buggi et al.^33^ also found mismatches in ER and PR status, tumor grade, proliferative index (Ki‐67), and HER‐2 status, in which 14 (12.4%) patients received different adjuvant treatments.

In contrast, Hilton et al.^34^ used several methods to measure tumor size; however, using alternative methods to measure tumor size did not provide additional prognostic information to treat patients with early‐stage breast cancer. Kanumuri et al.^19^ found that the histology and receptor status of the primary and secondary foci were highly consistent. Hence, they support a selective rather than a universal examination of each focus. East et al.^18^ supported the guidelines recommending that additional foci be tested if they are of different histology or grade. In our study, among the 10 patients with molecular subtype heterogeneity, only three had additional foci of different histology or grade.

The results of our study further confirm that the aggregate dimension has advantages in staging MF/MC breast cancer and in determining therapeutic methods. We also focused on the differences in pathologies and molecular types of different lesions, which may help us provide a more appropriate treatment for patients.

Over the decades, the definition of MF and MC breast cancers has not reached a worldwide consensus and has varied in different studies. The classic definition is based on the anatomical quadrant of the breast, which is divided by the clock position (3:00, 6:00, 9:00, or 12:00). Some scholars used anatomical quadrants,^35^ but the definition of quadrants was not mentioned in multiple studies. Alternatively, they used the tissue distance between each lesion to differentiate between MF and MC breast cancers. The distance between lesions was defined as 5 mm–5 cm.^16^, ^36^, ^37^ Some scholars have demonstrated that all the lesions are invasive tumors and that the tissue between each lesion must be benign,^33^, ^38^ whereas others included carcinoma in situ into the definition.^25^ When all lesions are invasive tumors, tissue between each lesion can be carcinoma in situ.^30^, ^39^ Previous studies mainly combined MF and MC breast cancers because of the ambiguity in their definition and the difficulty in distinguishing them.^32^, ^40^ In our study, we followed the classic definition with no distance specification so that more cases of MF/MC cancers could be studied to learn their special biological characteristics. We included carcinomas in situ because they are independent lesions in preoperative MRI evaluation, which was later confirmed by pathology. However, without following the anatomical method to divide the quadrant, we defined two foci as being in the same quadrant when they were connected to the nipple at an angle of <90°.

Our study used a retrospective study design that could have some sources of bias when collecting patients' clinicopathological data because the levels of pathology, imaging detection, and medical record systems 10 years previously were lower than those at present. Thus, a prospective study is required to obtain more precise results. The sample size also needs to be expanded to enhance the generalizability of our findings. To date, few studies have concentrated on MF/MC breast cancers despite their high incidence rates. Our study may change the inherent view of MF/MC breast cancers, and assist clinicians in developing more effective treatments for patients with MF/MC breast cancers. We are currently collecting more patient information and conducting follow‐up studies. We hope that this study can guide clinicians in the treatment of patients with MF/MC breast cancer.

In conclusion, the comparison of the two methods for measuring MF/MC tumor size reveals that the tendency of breast tumors to metastasize can be related to the total tumor load, which can be better predicted by the aggregate dimensions of all foci. The use of the current staging systems may require further evaluation and modification. Moreover, intertumoral heterogeneity can influence treatment strategies and outcomes. Therefore, pathological and immunohistochemical assessment of each lesion in patients with MF/MC breast cancer is essential.

## AUTHOR CONTRIBUTIONS

**Ying Tong:** Conceptualization (equal); data curation (equal); formal analysis (lead); investigation (equal); methodology (equal); resources (lead); software (equal); writing – original draft (lead). **Feixiang Sun:** Data curation (equal); formal analysis (equal); investigation (equal); resources (equal); software (equal); writing – original draft (equal). **Chuanpeng Zhang:** Data curation (equal); investigation (equal); software (equal). **Susu Yang:** Investigation (equal); resources (equal). **Ziyi Yu:** Conceptualization (equal); project administration (equal); supervision (equal); visualization (equal); writing – review and editing (equal). **Yi Zhao:** Conceptualization (equal); funding acquisition (lead); project administration (lead); supervision (lead); validation (lead); visualization (equal); writing – review and editing (equal).

## CONFLICT OF INTEREST STATEMENT

All authors indicated no potential conflicts of interest.

## ETHICAL APPROVAL

This study was conducted in accordance with the principles of the Declaration of Helsinki and was approved by the Ethics Committee of the First Affiliated Hospital of Nanjing Medical University (no: 2022‐SR‐293). This was a retrospective study; therefore, an exempt of written informed consent was granted by the ethics committee for this purpose.

## Data Availability

The data that support the findings of this study are available from the corresponding author upon reasonable request.

## References

[1] Sung H , Ferlay J , Siegel RL , et al. Global cancer statistics 2020: GLOBOCAN estimates of incidence and mortality worldwide for 36 cancers in 185 countries. CA Cancer J Clin. 2021;71(3):209‐249. 33538338 10.3322/caac.21660

[2] Girardi V , Carbognin G , Camera L , et al. Multifocal, multicentric and contralateral breast cancers: breast MR imaging in the preoperative evaluation of patients with newly diagnosed breast cancer. Radiol Med. 2011;116(8):1226‐1238. 21744256 10.1007/s11547-011-0704-7

[3] Houssami N , Ciatto S , Macaskill P , et al. Accuracy and surgical impact of magnetic resonance imaging in breast cancer staging: systematic review and meta‐analysis in detection of multifocal and multicentric cancer. J Clin Oncol. 2008;26(19):3248‐3258. 18474876 10.1200/JCO.2007.15.2108

[4] Berg WA , Gilbreath PL . Multicentric and multifocal cancer: whole‐breast US in preoperative evaluation. Radiology. 2000;214(1):59‐66. 10644102 10.1148/radiology.214.1.r00ja2559

[5] Coombs NJ , Boyages J . Multifocal and multicentric breast cancer: does each focus matter? J Clin Oncol. 2005;23(30):7497‐7502. 16234516 10.1200/JCO.2005.02.1147

[6] Bendifallah S , Werkoff G , Borie‐Moutafoff C , et al. Multiple synchronous (multifocal and multicentric) breast cancer: clinical implications. Surg Oncol. 2010;19(4):e115‐e123. 20615686 10.1016/j.suronc.2010.06.001

[7] Jain S , Rezo A , Shadbolt B , Dahlstrom JE . Synchronous multiple ipsilateral breast cancers: implications for patient management. Pathology. 2009;41(1):57‐67. 19089741 10.1080/00313020802563502

[8] Chua B , Ung O , Taylor R , Boyages J . Frequency and predictors of axillary lymph node metastases in invasive breast cancer. ANZ J Surg. 2001;71(12):723‐728. 11906387 10.1046/j.1445-1433.2001.02266.x

[9] Crowe JP Jr , Gordon NH , Shenk RR , Zollinger RM Jr , Brumberg DJ , Shuck JM . Primary tumor size. Relevance to breast cancer survival. Arch Surg. 1992;127(8):910‐915. discussion 915–6. 1642535 10.1001/archsurg.1992.01420080044007

[10] Cserni G , Chmielik E , Cserni B , Tot T . The new TNM‐based staging of breast cancer. Virchows Arch. 2018;472(5):697‐703. 29380126 10.1007/s00428-018-2301-9

[11] Giuliano AE , Connolly JL , Edge SB , et al. Breast cancer‐major changes in the American joint committee on cancer eighth edition cancer staging manual. CA Cancer J Clin. 2017;67(4):290‐303. 28294295 10.3322/caac.21393

[12] You KY , Zou WL , Ding L , Bi ZF , Yao HR . Large tumor size is an indicator for the timely Administration of Adjuvant Radiotherapy in luminal breast cancer with positive lymph node. Cancer Manag Res. 2021;13:1325‐1332. 33603478 10.2147/CMAR.S293470 PMC7884945

[13] Boros M , Marian C , Moldovan C , Stolnicu S . Morphological heterogeneity of the simultaneous ipsilateral invasive tumor foci in breast carcinoma: a retrospective study of 418 cases of carcinomas. Pathol Res Pract. 2012;208(10):604‐609. 22926109 10.1016/j.prp.2012.07.005

[14] Weissenbacher TM , Zschage M , Janni W , et al. Multicentric and multifocal versus unifocal breast cancer: is the tumor‐node‐metastasis classification justified? Breast Cancer Res Treat. 2010;122(1):27‐34. 20454925 10.1007/s10549-010-0917-9

[15] Boros M , Ilyes A , Nechifor Boila A , Moldovan C , Eniu A , Stolnicu S . Morphologic and molecular subtype status of individual tumor foci in multiple breast carcinoma. A study of 155 cases with analysis of 463 tumor foci. Hum Pathol. 2014;45(2):409‐416. 24439228 10.1016/j.humpath.2013.10.006

[16] Panuta A , Radu I , Gafton B , et al. Multiple versus unifocal breast cancer: clinicopathological and immunohistochemical differences. Rom J Morphol Embryol. 2019;60(1):103‐110. 31263833

[17] Pekar G , Gere M , Tarjan M , Hellberg D , Tot T . Molecular phenotype of the foci in multifocal invasive breast carcinomas: intertumoral heterogeneity is related to shorter survival and may influence the choice of therapy. Cancer. 2014;120(1):26‐34. 24127317 10.1002/cncr.28375

[18] East EG , Pang JC , Kidwell KM , Jorns JM . Utility of estrogen receptor, progesterone receptor, and HER‐2/neu analysis of multiple foci in multifocal ipsilateral invasive breast carcinoma. Am J Clin Pathol. 2015;144(6):952‐959. 26573003 10.1309/AJCPFWXP54OLILMU

[19] Kanumuri P , Hayse B , Killelea BK , Chagpar AB , Horowitz NR , Lannin DR . Characteristics of multifocal and multicentric breast cancers. Ann Surg Oncol. 2015;22(8):2475‐2482. 25805233 10.1245/s10434-015-4430-6

[20] Tot T , Gere M , Pekar G , et al. Breast cancer multifocality, disease extent, and survival. Hum Pathol. 2011;42(11):1761‐1769. 21663941 10.1016/j.humpath.2011.02.002

[21] Allison KH , Hammond MEH , Dowsett M , et al. Estrogen and progesterone receptor testing in breast cancer: ASCO/CAP guideline update. J Clin Oncol. 2020;38(12):1346‐1366. 31928404 10.1200/JCO.19.02309

[22] Wolff AC , Hammond MEH , Allison KH , et al. Human epidermal growth factor receptor 2 testing in breast cancer: American Society of Clinical Oncology/College of American Pathologists clinical practice guideline focused update. J Clin Oncol. 2018;36(20):2105‐2122. 29846122 10.1200/JCO.2018.77.8738

[23] Goldhirsch A , Winer EP , Coates AS , et al. Personalizing the treatment of women with early breast cancer: highlights of the St Gallen international expert consensus on the primary therapy of early breast cancer 2013. Ann Oncol. 2013;24(9):2206‐2223. 23917950 10.1093/annonc/mdt303 PMC3755334

[24] Hlawatsch A , Teifke A , Schmidt M , Thelen M . Preoperative assessment of breast cancer: sonography versus MR imaging. AJR Am J Roentgenol. 2002;179(6):1493‐1501. 12438043 10.2214/ajr.179.6.1791493

[25] Kelemen G , Farkas V , Debrah J , et al. The relationship of multifocality and tumor burden with various tumor characteristics and survival in early breast cancer. Neoplasma. 2012;59(5):566‐573. 22668023 10.4149/neo_2012_073

[26] Onisai M , Dumitru A , Iordan I , et al. Synchronous multiple breast cancers‐do we need to reshape staging? Medicina (Kaunas). 2020;56(5):230. 32403360 10.3390/medicina56050230 PMC7279247

[27] Curigliano G , Burstein HJ , Winer EP , et al. De‐escalating and escalating treatments for early‐stage breast cancer: the St. Gallen International Expert Consensus Conference on the primary therapy of early breast cancer 2017. Ann Oncol. 2017;28(8):1700‐1712. 28838210 10.1093/annonc/mdx308 PMC6246241

[28] Andea AA , Wallis T , Newman LA , Bouwman D , Dey J , Visscher DW . Pathologic analysis of tumor size and lymph node status in multifocal/multicentric breast carcinoma. Cancer. 2002;94(5):1383‐1390. 11920492 10.1002/cncr.10331

[29] Boyages J , Jayasinghe UW , Coombs N . Multifocal breast cancer and survival: each focus does matter particularly for larger tumours. Eur J Cancer. 2010;46(11):1990‐1996. 20385484 10.1016/j.ejca.2010.03.003

[30] Boros M , Podoleanu C , Georgescu R , Moldovan C , Molnar C , Stolnicu S . Multifocal/multicentric breast carcinomas showing intertumoural heterogeneity: a comparison of histological tumour type and Nottingham histological grade of primary tumour and lymph node metastasis. Pol J Pathol. 2015;66(2):125‐132. 26247525 10.5114/pjp.2015.53008

[31] Salgado R , Aftimos P , Sotiriou C , Desmedt C . Evolving paradigms in multifocal breast cancer. Semin Cancer Biol. 2015;31:111‐118. 25066860 10.1016/j.semcancer.2014.07.002

[32] Fushimi A , Yoshida A , Yagata H , et al. Prognostic impact of multifocal and multicentric breast cancer versus unifocal breast cancer. Surg Today. 2019;49(3):224‐230. 30317491 10.1007/s00595-018-1725-9

[33] Buggi F , Folli S , Curcio A , et al. Multicentric/multifocal breast cancer with a single histotype: is the biological characterization of all individual foci justified? Ann Oncol. 2012;23(8):2042‐2046. 22219015 10.1093/annonc/mdr570

[34] Hilton JF , Bouganim N , Dong B , et al. Do alternative methods of measuring tumor size, including consideration of multicentric/multifocal disease, enhance prognostic information beyond TNM staging in women with early stage breast cancer: an analysis of the NCIC CTG MA.5 and MA.12 clinical trials. Breast Cancer Res Treat. 2013;142(1):143‐151. 24113743 10.1007/s10549-013-2714-8

[35] Shaikh T , Tam TY , Li T , et al. Multifocal and multicentric breast cancer is associated with increased local recurrence regardless of surgery type. Breast J. 2015;21(2):121‐126. 25597248 10.1111/tbj.12366

[36] Duraker N , Caynak ZC . Axillary lymph node status and prognosis in multifocal and multicentric breast carcinoma. Breast J. 2014;20(1):61‐68. 24438065 10.1111/tbj.12205

[37] Ilic IR , Petrovic A , Zivkovic VV , et al. Immunohistochemical features of multifocal and multicentric lobular breast carcinoma. Adv Med Sci. 2017;62(1):78‐82. 28189947 10.1016/j.advms.2016.07.003

[38] Ustaalioglu BO , Bilici A , Kefeli U , et al. The importance of multifocal/multicentric tumor on the disease‐free survival of breast cancer patients: single center experience. Am J Clin Oncol. 2012;35(6):580‐586. 21926901 10.1097/COC.0b013e31822d9cd6

[39] Kuan LL , Tiong LU , Parkyn R , Walters D , Lai C , Walsh D . Disease recurrence and survival in patients with multifocal breast cancer: a follow‐up study with 7‐year results. ANZ J Surg. 2017;87(10):E125‐E128. 26074155 10.1111/ans.13193

[40] McCrorie AD , Ashfield S , Begley A , et al. Multifocal breast cancers are more prevalent in BRCA2 versus BRCA1 mutation carriers. J Pathol Clin Res. 2020;6(2):146‐153. 32022473 10.1002/cjp2.155 PMC7164372

